# The Generation of Forces and Moments during Visual-Evoked Steering Maneuvers in Flying *Drosophila*

**DOI:** 10.1371/journal.pone.0004883

**Published:** 2009-03-20

**Authors:** Hiroki Sugiura, Michael H. Dickinson

**Affiliations:** 1 Japan Aerospace Exploration Agency, Chofu, Tokyo, Japan; 2 California Institute of Technology, Pasadena, California, United States of America; The Rockefeller University, United States of America

## Abstract

Sideslip force, longitudinal force, rolling moment, and pitching moment generated by tethered fruit flies, *Drosophila melanogaster*, were measured during optomotor reactions within an electronic flight simulator. Forces and torques were acquired by optically measuring the angular deflections of the beam to which the flies were tethered using a laser and a photodiode. Our results indicate that fruit flies actively generate both sideslip and roll in response to a lateral focus of expansion (FOE). The polarity of this behavior was such that the animal's aerodynamic response would carry it away from the expanding pattern, suggesting that it constitutes an avoidance reflex or centering response. Sideslip forces and rolling moments were sinusoidal functions of FOE position, whereas longitudinal force was proportional to the absolute value of the sine of FOE position. Pitching moments remained nearly constant irrespective of stimulus position or strength, with a direction indicating a tonic nose-down pitch under tethered conditions. These experiments expand our understanding of the degrees of freedom that a fruit fly can actually control in flight.

## Introduction

Flying insects display stability, maneuverability, and robustness that are rarely matched by either fixed- or rotary-wing aircraft. Such performance has prompted interest in insect flight, both as a model system for sensory motor integration [1]–[2] and as a means of inspiration for developing new control algorithms for technological devices [3]–[6]. Although our understanding is still limited, recent studies with fruit flies, locusts, and other insects have begun to uncover how insects manipulate time-varying forces to implement flight control algorithms [7]–[9].

Previous measurements of forces and moments in flies have focused on the control of thrust and lift [10]–[13], which co-vary in *Drosophila* [14], or yaw torque [11], [15]–[17]. Blondeau and Heisenberg [18] separately measured yaw, pitch, and roll torques generated by fruit flies, and found that flies make compensatory reactions in response to rotatory visual motions around all three axes. One important feature of flight control algorithms is the degree to which animals accomplish maneuvers through coordinated changes in multiple output degrees of freedom [7]. For example, a fly might accomplish a simple avoidance reaction through a change in yaw, roll, sideslip, or a combined change in multiple forces and moments. Studies of blow flies suggest that the control of forces and moments are tightly coupled through the influence that individual steering muscles have on wing kinematics [19]. Our approach here is to directly measure the forces and torques generated by tethered fruit flies in response to translational patterns of optic flow and to correlate them with observed changes in wing kinematics. The results indicate that the fruit flies are capable of generating sideslip and that visually-elicited turning responses involve a coordinated change in both forces and moments.

## Materials and Methods

### Animals

We used one- to three-day-old female fruit flies (*Drosophila melanogaster*, *M.*) collected from a laboratory colony that originated from a mixture of 200 wild-caught females. The flies were reared at low density in bottles so that females emerged at large body size and low variability. Individuals were cold-anesthetized and tethered to a tungsten rod with UV-activated glue as has been described previously [20]. Each fly was tethered perpendicularly to a tungsten rod to the notum at the anterior end of the thorax.

### Electronic flight arena

**Figure 1:**
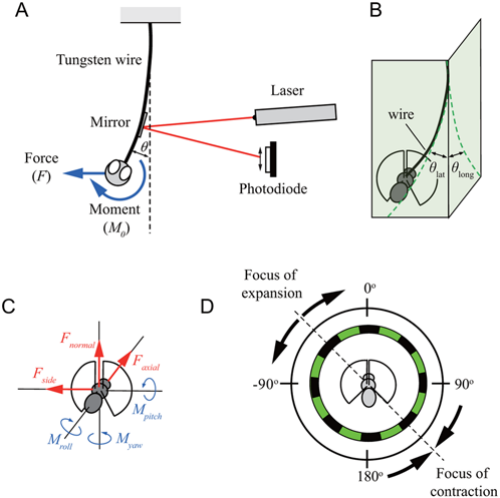
Schematic of an angular deflection of a tethered wire. (A) 2-D schematic. (B) 3-D schematic. (C) Six force components and point of origin of the fly. (D) Schematic of the experimental setup.

Experiments were conducted within a cylindrical flight arena consisting of 96 columns and 36 rows of light-emitting diodes (LEDs) [21]. Each LED subtended approximately 3.75°. For these experiments we created translational patterns consisting of square wave gratings that moved in opposite directions on two sides of the arena creating a focus of expansion (FOE) and a focus of contraction (FOC) spaced 180° apart (Fig. 1D). The luminance of the bright and dark panels was 72 and 2.7 cd m^−2^, respectively, and the Michelson contrast was 93%. A more detailed description of the display panels and their operation is provided elsewhere [21]. The spatial wavelength of each square wave was 30° and the angular velocity of the pattern was 150°s^−1^, corresponding to a temporal frequency of 5 s^−1^. A temporal frequency of 5 s^−1^ was chosen for the expanding stimulus because it elicits a maximum turning response as measured in a recent behavioral study [22]. To map the directional response of each animal, we rotated the azimuthal position of the FOE (and thus the FOC) in random order. Each visual pattern lasted 3 s and was followed by a 2 s rest period in which the pattern was stationary.

The pattern of contraction and expansion that we programmed into the display is a coarse simplification of the optic flow pattern that an insect would experience if it were to translate in free flight. In particular, our pattern consists exclusively of azimuthal motion of vertical bars, whereas a true translatory stimulus would include oblique and vertical components. It is possible that a more naturalistic stimulus might elicit a stronger behavioral response than we have detected. However, a recent study on responses to rotatory and translatory flow patterns, which used exactly the same stimuli as we have employed [22], suggest that such patterns elicit very robust and perhaps saturating responses. Nevertheless, while for convenience we use the terms FOE and FOC throughout the paper, we define them only as rough approximations of true translatory flow.

### Optical force measurements

The largest forces generated by fruit flies, roughly 150% of body weight (15 µN), are still quite small in absolute terms [20], and sideslip forces are likely to be much smaller. Commercially available force sensors based on piezoelectric devices or strain gauges are capable of measuring a few micronewtons at best [23]. Sun and co-workers [24] describe a novel multi-axis force sensor based on MEMS technology, but such devices are not yet in commercial production. The resolution of the optical method we employed was roughly 0.1 µN. All forces were measured optically by tracking the deflection of the tungsten rod to which the fly was tethered. A 2 mm×2 mm×0.1 mm thick mirror was fixed to the rod, and the angular deflections were measured by aiming a 5 mW red diode laser at the mirror and tracking the reflected beam using a position-sensitive differential photodiode (SL5-2, United Detector Technologies, Hawthorne, California) (Fig. 1A). The photodiode output signal was amplified and filtered (low-pass) at 10 Hz prior to digital conversion using Digidata hardware (Axon Instruments) and a PC running Axoscope software. An 18° opening in the rear of the cylindrical display provided an unobstructed path for the incident and reflected beam. For side force and rolling moment measurements, data from two experiments with the opening on either the right or left were averaged to exclude setup asymmetry. The distance between the mirror and the photodiode was sufficiently large so that the vertical displacement of the reflected laser spot was linearly proportional to the angular deflection of the wire.

The key to interpreting the deflections generated by the fly is in separating the contributions of forces and moments applied by the fly at the tip. The angular deflection, *θ*, of a thin beam of length, *l*, is related to the force, *F*, and moment, *M*_0_, applied at its tip by (Fig. 1A):
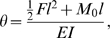
(1)where *E* is Young's modulus for tungsten and *I* is the moment of inertia of the cross-sectional area of the wire. This derivation assumes that the deflection of the wire is small so that small angle approximations apply. In this experiment, *F* is the resultant of side force, *F_side_*, and axial force, *F_axial_*, and *M*_0_ is the resultant magnitude of pitching moment, *M_pitch_*, and rolling moment, *M_roll_*. The lateral angular deflection of the wire, *θ_lat_*, is defined as the angular deflection of a rearward projection of the wire (Fig. 1B):
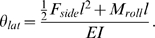
(2)The longitudinal angular deflection, *θ_long_*, is defined as the angular deflection of a lateral projection of the wire (Fig. 1B):
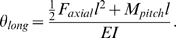
(3)The assumption of a small angular deflection in this experiment was justified by estimated values of the measured angular deflection of *θ_lat_* and *θ_long_*, which were less than 8×10^−5^ and 8×10^−4^ rad, respectively.

We used different wires for pitch-and-axial-force and roll-and-side-force measurements, respectively. The magnitude of the axial force is presumably more than 10 times larger than that of side force. Thus, in order to measure the very small side force, we used more sensitive wires. We chose less sensitive wire for axial-force measurement to maintain response linearity within the realm of our measurements.

For the sake of simplicity, an orthogonal reference system was chosen in which rotational axes coincided with translational axes (Fig. 1C), similar to the conventions adopted previously [15]. The coordinate system is standard for insect flight mechanics, but is not the most intuitive one for interpreting maneuvers since yaw does not correspond to functional yaw of the animal, which is perpendicular to the horizontal plane [25]. For example, to generate a change in heading (a pure functional yaw) the fly would have to generate a combination of roll and yaw moments. The same is true for axial and normal forces. Functional thrust, which is aligned to the forward flight direction, lies in the same plane as the axial and normal forces, but differs from both force components. The tethered point was defined as the origin of our coordinate system. The names of force components were derived from the vocabulary used in most texts on aircraft flight mechanics [26]. Accordingly, the sideward/sideslip force component is called side force, and perpendicular and longitudinal force components are defined as normal and axial forces, respectively.

### Force composition

As shown in Eqs. 2 and 3, the contributions of force and moment on angular deflection of the rod depend on the rod's length. This dependency allowed us to separate the contributions of forces and moments by conducting a series of measurements using rods of different lengths. Because we could not perform a complete set of measurements of a single fly (i.e. un-tether and re-tether an animal to a longer rod), this technique assumes that the directional tuning responses of different flies are similar – an assumption that is supported by previous behavioral studies [17]. We used three wires instead of two for the detection of the smaller side force, which decreased our measurements error. Standard deviation of the side force using two equations was 0.25 micronewtons and that using three equations was 0.15 micronewtons.

We should note that our methods must assume that the forces and moments generated by the different populations of flies used for each wire length are equivalent. The most likely way for this assumption to be violated is if the groups of flies assigned to each wire length differed significantly in body mass, a value that correlates strongly with total flight force [20]. We did not weigh flies at the onset of the experiment, because this would have required a harsher anesthesia (e.g. CO_2_), which decreases the performance of flies in the flight arena. Measuring flies after each experiment is problematic, because they loose weight in a time-dependent fashion that is not easy to control. However, we could assess the likelihood of errors due to weight differences by examining the population variability within our experimental groups. We measured body weight for individuals randomly picked from the fly stocks reared under the same low density condition as was used for the force measurement (1.16+/−0.099 mg, mean+/−S.D., N = 96). We used 12 flies for each of our wire lengths in pitch and axial-force measurements, and a minimum of 20 flies for the roll and side-force measurements. The estimated deviation is given by the standard error, which is 0.0284 mg (2.45% body weight) for pitch and axial force and 0.0220 mg (1.90% body weight, calculated with N = 20) for roll and side slip force. Assuming that force scales in proportion to body mass, the difference in the force should be 2.45% for the pitch measurement and 1.90% for the roll measurement, both of which are much smaller than the measured errors of all the forces and moments. Thus, we conclude that variation in body size between populations used for different wire lengths did not make a substantial contribution to our total measured error.

Our measurement scheme provides us with values for moments measured around the tip of the wire, whereas values about the fly's center of mass are more informative. To calculate the moment about the fly's center of mass, we implemented the parallel axis theorem. The fly's body was assumed rigid, and the center of mass was assumed to lie in the mid-sagittal plane, and the center of pressure of the wing was located at a 70% semi-span position. The value for the span-wise center of pressure was based on both computational [27] and experimental measurements of the distribution of chord-wise circulation along the wing [28]–[29]. Similar assumptions have been adopted in previous studies [8], [30].

### Calibration and selection of wire

**Figure 2:**
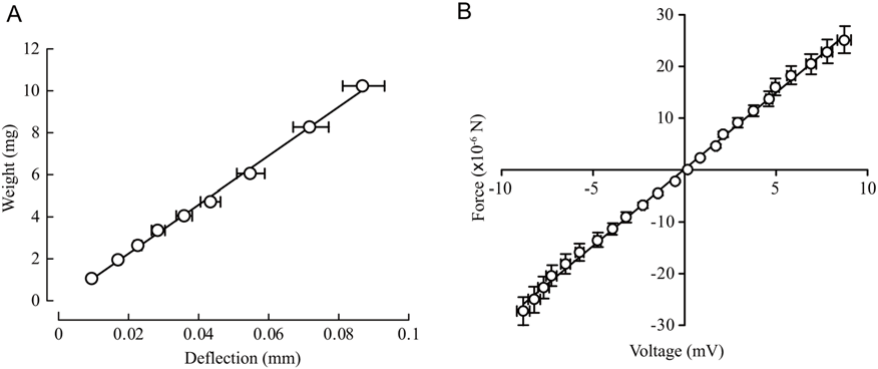
Weight-to-deflection curves for the 19.1 mm-long wire. (A) Force-to-deflection calibration of the wire as the first step of posteriori calibration. (B) Force-to-output-voltage curve for the whole calibration for the wire.

Calibration of our sensor method was carried out using two steps. First, a static force-to-deflection calibration was conducted using a set of weights constructed from aluminum foil strips. We aligned the wire horizontally and measured the deflections using a 60× stereomicroscope. For small forces, the deflection of a horizontal wire will be equal to that of a vertically-aligned wire. Second, a deflection-to-diode voltage calibration was carried out by translating a rod attached to the wire using a micromanipulator equipped with a calibrated micrometer. Table 1 shows the length and diameter of all wire beams used in the experiments. We used a total of 40 different wires, 8 for each length. Fig. 2A shows the weight-to-deflection curve of the first step of the calibration for 19.1 mm-long wire, and Fig. 2B shows the force-to-output voltage curve for the whole wire calibration.

**Table 1:** Length and diameter of the tungsten wires.

Wire no.	length (mm)	diameter (mm)	resonance frequency (Hz)	corresponding equation
I	24.8	0.10	330	(3)
II	16.5	0.10	400	(3)
III	19.1	0.20	110	(2)
IV	15.2	0.20	120	(2)
V	12.7	0.20	140	(2)

The resonance frequency of the tungsten rod was an important constraint for our measurement system. We determined the resonance frequency of the wire by gently flicking it using a human hair with a dead, but undesiccated, fly attached. We used only wires with resonance frequencies above 100 Hz, which is ten times greater than the cut-off of our analog low-pass filter. To limit complications of resonance, we used wires that had resonant frequencies either 1.5 times higher or 0.67 times lower than the 220 Hz wing beat frequency typical of most flies. We empirically judged that this window was sufficient to ensure that the motion of the fly was not amplified by system dynamics. Unless stated otherwise, all data values for forces and moments are presented as means+/−uncertainty at 95% confidence level.

## Results

**Figure 3:**
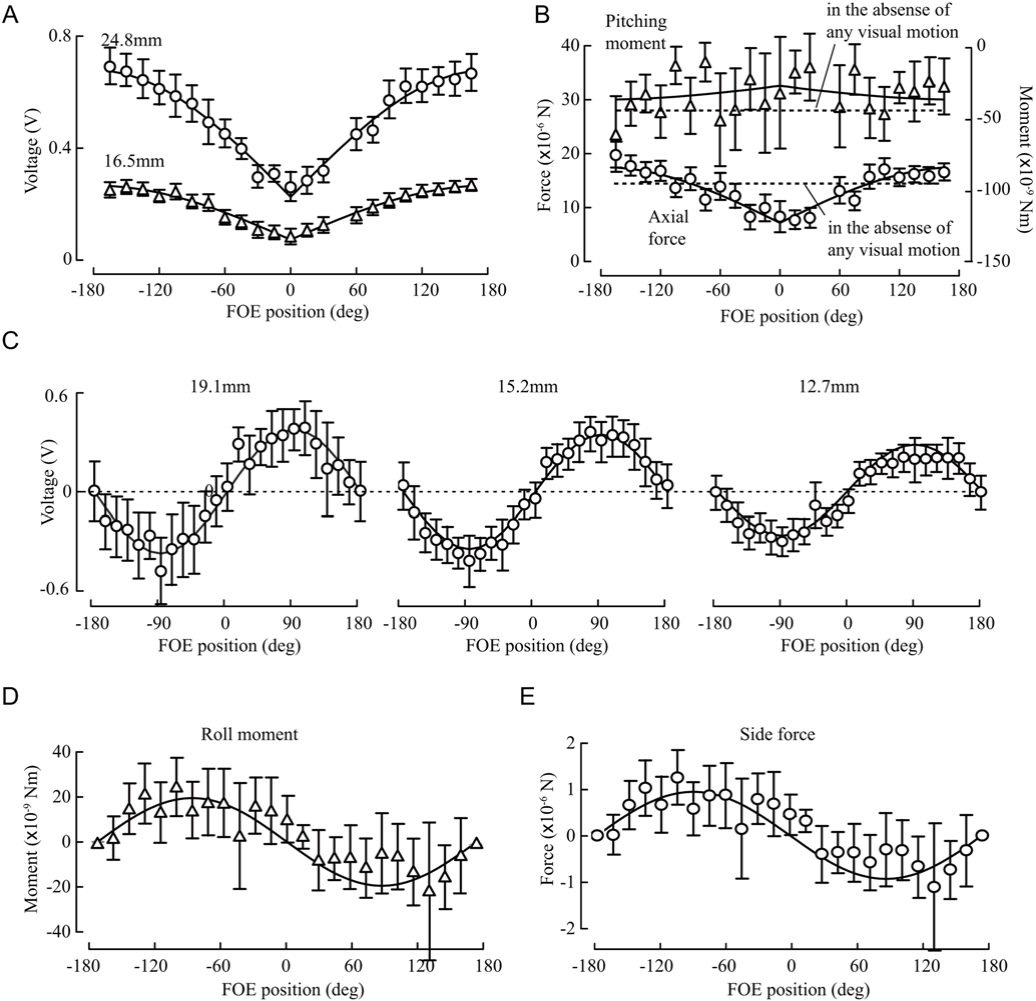
Flies' response to changes in FOE position: axial force, pitch, side force and roll. (A) Mean voltage vs. FOE position for axial-force measurement using two different wire lengths. (B) Axial force and pitching moment of tethered flies vs. the FOE position. (C) Mean voltage vs. the FOE position for side-force measurements using three different wire lengths. (D) Rolling moments vs. the FOE positions. (E) Side forces vs. the FOE positions.

Figure 3A shows the flies' responses to changes in the azimuthal position of the focus of expansion, plotted in terms of absolute voltage changes measured using 24.8 mm- and 16.5 mm-long wires (*N* = 12). The data are fit with a sine function for the absolute value of a FOE position. In both cases, the data are in very good agreement with these simple arbitrary functions. A set of simultaneous equations (using equation 3, after converting voltages to deflection) was created from the voltage data for each wire length at each FOE position, which allowed us to solve for values of axial force and pitching moment for each position by least squares (Fig. 3B). As with the raw voltage values, the axial force and moment data were reasonably well fit by a sine function of the absolute value of FOE position. Axial force was minimal (7×10^−6^±3×10^−6^ N) when the FOE was directly in front of the fly, and maximal (17×10^−6^±2×10^−6^ N) when the FOE was directly behind the fly. The pitching moment varied little with FOE position. We calculated the moment around the center of mass to be −60×10^−9^±40×10^−9^ Nm and −80×10^−9^±20×10^−9^ Nm at the 0° and 180° FOE positions, respectively. The sign convention is such that negative values indicate nose-down pitch. This means that tethered animals generate a large tonic amount of nose-down pitch, confirming results in a prior study in which forces were derived from wing kinematics [8].

The comparable results for side force and rolling moment measurements are shown in Figs. 3C–E. To maintain measurement accuracy for detection of smaller side-slip forces, we used three wire lengths and increased our sample size when using the shortest, least sensitive wires. Figure 3C plots the mean voltage against FOE position using wires with three different length, 19.1 mm (*N* = 20), 15.2 mm (*N* = 24), and 12.7 mm (*N* = 28). A sine curve was fitted to the data for each wire, which show good agreement. As with pitch and axial force, we used the voltage data at each FOE position to calculate roll and side force according to Eq. 2. (Figs. 3D, E). In this case, however, we used a set of three simultaneous equations. The relationship between rolling moment and FOE position was sinusoidal (Fig. 3D), indicating that the fly actively modulates roll in response to a translational stimulus in the horizontal plane. The sign of the response is such that the animal rolls away from an expanding visual field. The relationship between side force and FOE position was also sinusoidal, with flies attempting to slip away from the FOE (Fig. 3E). The peak force, generated when the FOE was at ±90°, was 0.8×10^−6^±0.5×10^−6^ N, or roughly 8% of body weight. Because the flies were tethered, it is clear that *D. melanogaster* is capable of modulating side force without changing its body orientation.

## Discussion

**Figure 4:**
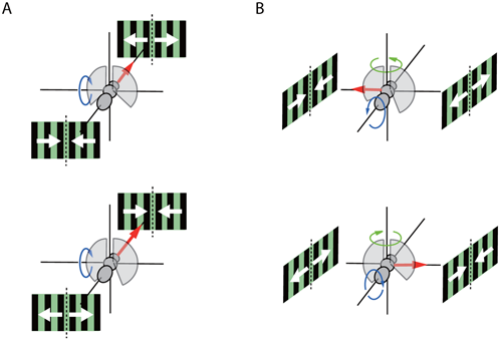
Cartoon summary of results. (A) Flies increase axial force and maintain downward pitch in response to a forward FOC. (B) Flies attempt to move away from a lateral FOE with a combined sideslip and roll.

The forces and moments generated by tethered fruit flies in response to visual stimuli were measured to provide insight into the behavioral and aerodynamic mechanisms of flight control. Our results show that a fruit fly's reaction to a translational visual stimulus involves a coordinated modulation of forces and moments. In particular, in response to a lateral FOE, which simulates sideways motion, an animal generates side-slip force and roll away from the expansion (Fig. 3D, E; 4B). Both reactions would accelerate the animal away from the expanding stimulus. Tammero et al. [17] reported the yawing-away response consistent with these reactions which is indicated by green arrows in the Fig. 4B. In response to a FOE directly in front of an animal, a flow pattern that simulates forward motion, a tethered animal decreases axial force and maintains a nose down pitch (Fig. 3B; 4A).

These measurements were possible by developing a multi-wire technique that allowed us to separate forces and moments created by a fly tethered to a fine beam. We can assess the accuracy of our measurements by comparison with some prior studies. We measured a maximum axial force value of 17×10^−6^±2×10^−6^ N, which is consistent with measurements by Götz [11], who reported a value of 14×10^−6^±4×10^−6^ N. Götz [11] used an electro-inductive means to measure the force, which was entirely different from the optical method employed here. We measured a maximum rolling moment about the center of mass of 20×10^−9^±8×10^−9^ Nm in response to an expanding visual pattern, which is about 2/3 of the peak value reported by Blondeau and Heisenberg [18] for a roll moment generated in response to a rotatory visual stimulus around the roll axis. Our roll moment was similar in magnitude to the yaw moment reported in response to an expanding stimulus [17]. This prior observation suggests that flies respond to a lateral expansion with a change in both roll and yaw moments of comparable magnitude.

In addition to generating strong rolling and yawing moments, flies also modulated side slip in response to a translational visual stimulus, producing a peak force when the FOE was lateral (±90°). The peak force was 0.8×10^−6^±0.5×10^−6^ N, or roughly 8% of body weight. The observed side force modulation is not a result of simply rolling the thorax to tilt the mean stroke plane. Even when producing maximum lift, roughly 150% of body weight, the fly would need to roll away by about 5° in order to attain the magnitude of the measured side force. This degree of tilt is impossible in our tethered configuration; we estimated the amount of permissible roll to be less than 5×10^−4^ °. If the fly were tethered with a skew of 5 degrees, the axial force would be resolved as a sideways component of this magnitude. However, as we can see from the Figures 3A and 3C, the sinusoidal response was directionally consistent and symmetrical on both sides of the y-axis. Accordingly, the alignment errors in tethering are small and much less than 5°. This observation of active side slip in tethered flies is consistent with a recent free flight observation in *Drosophila* [31]. In addition, Collet and Land [32] observed that the hoverfly, *Syritta pipiens*, can fly sideways without changing its heading, and Blondeau reported possible modulations of side force in *Calliphora*, which were roughly 8% of body weight [15], identical to the value we measured in *Drosophila*.

The tethered flies created a tonic nose-down pitch moment of approximately – 70×10^−9^±30×10^−9^ Nm (Fig. 3B). Based on high speed video kinematics replayed through a dynamically-scaled robot, Fry and co-workers [8] also reported this downward pitch, and showed that it is in some way an artifact of tethering because animals hovering in free flight create zero mean pitch. This strong pitch is correlated with the presence of clap and fling kinematics which shifts the point of wing pronation backward behind the center of mass. This creates downward pitching moment which is consistent with the present results. Using a mechanical model, Lehmann and Pick [33] stated that the clap and fling reinforce the pitching moment by upto 21%. However, free flying flies rarely use the clap and fling, even during take-off [31], [34], which suggests that the clap and fling and the resulting production of nose down pitch are an artifact of tethering. There are two likely explanations for this artifact. The first is that the tethering procedure changes the mechanics of the thorax, thereby distorting the wing stroke. The second possibility is that tethering might alter a fly's flight velocity control system. When on a tether, many sensory systems, such as the eyes, antennae, ocelli, and halteres, are not receiving the information they would in forward flight, deficits which might collectively trigger a reaction to accelerate forward by pitching downward to reorient the mean flight force vector [8]. The hypothesis is consistent with David's observation on the relationship between body orientation (pitch angle) and flight speed in *Drosophila hydei* [35]. It is interesting to note that even when the fly's body axis is positioned horizontally, as in the present experiment, it still attempts to pitch down, as if the animal's absolute angular orientation has little or no effect on its regulation of pitch moment.

Axial thrust is maximal when the focus of expansion (FOE) is behind the fly, as it would be if the fly was blown backward by a head wind. Peak sideslip was generated in response to a lateral FOE, suggestive of either a centering [36] or collision avoidance response [17]. The response to a forward pole of expansion is most likely part of a velocity control algorithm [37]. The similar response functions for sideslip, roll, and yaw suggest that the three behaviors are part of a single coordinated reflex that acts to carry the animal away from an impending lateral collision. This is in accordance with a report by Blondeau and Heisenberg [18] which suggested that the well known optomotor yaw response of *Drosophila* is part of a 3-dimensional optomotor torque system of roll, pitch and yaw.

Our data address, but do not resolve, the important question as to how many degrees of moment flies can control [7], [38]. The present method is incapable of detecting independent control of roll, yaw and side force. This is because the estimated forces and moments were calculated using sine functions fitted to the data and the forces and moments will necessarily be correlated. However, comparison of Figs. 3B, D and E demonstrates that the side force, axial force and pitch vary differently according to the same sensory input. This implies that flies can actively modulate side force, axial force and pitch independently. This means that *Drosophila* can control at least three degrees of freedom. They also control yaw and roll moments, but as discussed above, the present method is incapable of detecting that they can do so independently of side force. Further, Götz and Wandel [14] have already demonstrated that axial force (what they called thrust) strongly covaries with lift, suggesting that flies modulate the magnitude but not the orientation of the mean flight force in the mid-sagittal plane. Thus, all current evidence suggests that flies possess three output degrees of freedom: 1) pitch, 2) axial force/lift, and 3) side force/roll/yaw. It is still possible the fly can exert some independent control over the coupled components (e.g. side force, roll, and yaw), but there is no evidence yet that they do. Such evidence might emerge from the use of more complex force probes in tethered preparations or more detailed analysis of free flight trajectories coupled with an accurate dynamic model. Another important next step toward understanding flight control will be to determine how flies control their output degrees of freedom via changes in motor activity and wing kinematics, combing studies of aerodynamics, behavior and neurobiology [19]. With such additional data it should also be able to test whether the directional tuning of a fly's sensory systems are matched to the actuator modes of its motor system, as has been recently suggested [39].
